# The Origin and Evolution of the 18th Century Hospital Movement
*Previous articles appeared on December 13, 1913, January 17 and 31, February 14 and 28, and March 14, 1914.


**Published:** 1914-03-28

**Authors:** 


### VII. THE ORIGIN AND EVOLUTION OF THE 18th CENTURY HOSPITAL MOVEMENT.*

FOLLOWING closely upon the success obtained at Winchester, similar institutions were opened at Bristol and York, and while the fifth decade witnessed the steady progress of the movement in the Western counties, it was characterised by the foundation of the first "special" hospitals in London.

### THE FIRST LONDON HOSPITAL FOR MOTHERS.

As yet the London hospitals could be counted upon the fingers of one hand—St. Bartholomew's in Smithfield, St. Thomas's and Guy's in Southwark, and the two voluntary institutions at Westminster—and the inadequacy of their accommodation to the needs of the sick poor becomes wearisome by its repetition when consulting contemporary hospital literature. In 1740 the London Hospital was established to supply the wants of the eastern suburb, and five years later the Middlesex Infirmary opened its doors to the notoriously miserable denizens of St. Giles's; this latter having the distinction of being the first London charity to provide (in 1747) for the reception of lying-in women.

Although Bath had been visited occasionally for its waters by persons of the highest rank, it was not until the eighteenth century that it became the habitual resort of the fashionable world. Evidence exists that, as early as 1725, a proposal was circulated to erect an infirmary in that city "for the care and maintenance of the great concourse of poor strangers coming here for the use of the medicinal waters, and begging of the gentry here for their support." And although the wording suggests a recrudescence of those earlier motives which prompted the segregation of the sick poor for the protection of the wealthy, the Bath Infirmary is entitled to the distinction of having been the first *in conception* outside London.

### BEAU NASH AS A HOSPITAL OFFICER.

This and several subsequent attempts were, however, doomed to failure until, under the patronage of Richard (Beau) Nash, Mead, and several eminent physicians, the design was consummated by incorporation under the title of The President and Governors of the General Hospital or Infirmary at Bath, which was opened to the public in 1742.

The charter, while securing the use of the Baths to the poor, and prescribing the terms upon which patients should be admitted, stipulated that the charity should be open to suitable persons from all parts of his Majesty's dominions; the majority of the cases admitted being of a chronic nature—leprosies, palsies, old inveterate rheumatisms, and lamenesses, which had been treated by every other method elsewhere without success.

The Bath Hospital was the first to which the term *general hospital* was applied; and though in modern parlance those words are taken to mean an institution for the reception and treatment of all, or nearly all, forms of disease (as opposed to special hospitals, limited to one or more diseases), there remains no doubt that its original application was intended to denote that those general hospitals received patients from all parts. The diseases for which the Bath Hospital was intended were those which would be relieved or cured by its waters; they were special and limited, while the area from which patients were received was general and unlimited. Yet this was a general hospital.

Again, Addenbrooke's Hospital, both by the will of its founder and by the Act of Incorporation, was described as a general hospital for the poor of any parish or county; and Queen Charlotte's was known in 1764 (if not earlier) as the General Lying-in Hospital. Though limited to one special class of cases, the area from which it then received patients was general and unlimited.

### THE GROWTH OF SPECIAL HOSPITALS.

On the other hand, the term *special* as applied to hospitals was then unknown. Putting aside the Royal foundation of Bedlam, the first institutions for the treatment of one (only) disease were the Dublin Lying-in Hospital, opened in 1745, and the Middlesex County Hospital for Smallpox and Inoculation, in 1746, which latter was described as "a hospital in aid of other hospitals, since it receives those whom the rules of all other charities expressly exclude." These words sound the keynote of the motives underlying the foundation of all the early hospitals for the treatment of any one single disease: which motives differed from those which suggested the provision of the hospitals of the nineteenth century for diseases of the eyes, ears, etc., inasmuch as the latter were established, not for the reception of cases which would have been refused by the general hospitals (in the modern meaning), but for the purpose of providing special treatment for those diseases, in accordance with the then recent revival of that specialism in medicine which, thousands of years before, had been the prominent characteristic of Babylonian and Egyptian practice.

The Dublin Lying-in Charity, which was the first of its kind in his Majesty's dominions, is one of the most remarkable in the history of hospitals. Established at the sole instance and expense of Dr. Bartholomew Mosse, a physician of that city, it was not until he had devoted many years to its management, and had exhausted his private fortune in its maintenance, that he was assisted by the voluntary contributions of the public, and, later still, obtained large Parliamentary grants which enabled him to carry the project to efficient completion. In 1756 a charter was obtained; at the end of the following year the new (Rotunda) building was opened for the reception of patients, and Dr. Mosse was appointed its Master for life. He lived long enough to witness the consummation of his design, but, worn out by anxiety and hard work, he died in 1759, at the early age of forty-six. By his unaided efforts he had procured for the charity, initiated in a small dwelling-house, so favourable a reputation for successful treatment that particulars of its management were sought and obtained by other practitioners, who succeeded in establishing similar charities in London. Though the founder of an institution which, as hospital and school, has acquired world-wide fame and shed lustre upon his country, his name is well-nigh forgotten, except by those conversant with the details of hospital history. *Sic transit gloria mundi.*

NOTE.—*Our readers may also be referred to an illustrated article called "Beau Nash as a Hospital Officer," which appeared in* THE HOSPITAL *of March 7, p. 627.*

* Previous articles appeared on December 13, 1913, January 17 and 31, February 14 and 28, and March 14, 1914.

